# Antimicrobial and Nutritional Properties of Edible Oyster Mushroom (*Pleurotus ostreatus*) Extracts Against Foodborne Pathogens Relevant to Maternal and Child Malnutrition in Africa

**DOI:** 10.1002/fsn3.72003

**Published:** 2026-06-22

**Authors:** Abdirasak Sharif Ali, Yahye Ahmed Nageye, Kizito Eneye Bello

**Affiliations:** ^1^ Department of Microbiology and Laboratory Sciences, Faculty of Medicine and Health Sciences SIMAD University Mogadishu Somalia; ^2^ Department of Microbiology, Faculty of Natural Science Kogi State (Prince Abubakar Audu) University Anyigba Kogi State Nigeria

**Keywords:** antimicrobial activity, foodborne pathogens, functional foods, GC–MS, maternal and child nutrition, nutritional composition, *Pleurotus ostreatus*

## Abstract

*Pleurotus ostreatus* (oyster mushroom) is an edible mushroom with nutritional value and potential antimicrobial constituents, making it relevant to food‐quality research and to future maternal and child nutrition strategies in settings where diet quality and enteric infections intersect. This exploratory laboratory‐based experimental study assessed the antibacterial activity, nutritional composition, tentative chemical profile, and in silico target interactions of aqueous and ethanolic extracts of *P. ostreatus* obtained from Somalia. Aqueous and ethanolic extracts were evaluated by agar diffusion against *Staphylococcus aureus* , *Escherichia coli* , *Salmonella enterica* , *Shigella flexneri* , and *Listeria monocytogenes* ; MIC/MBC and time‐kill assays were performed for selected isolates. Nutritional composition was determined by standard proximate procedures; iron and zinc were measured by atomic absorption spectrophotometry; bioactive compounds were tentatively profiled by GC–MS using spectral‐library matching; and selected compounds were explored by molecular docking. At 200 mg/mL, the ethanolic extract produced inhibition zones of 18.2 ± 0.8 mm against *S. aureus* , 16.0 ± 0.7 mm against *E. coli* , 14.2 ± 0.6 mm against *S. enterica* , 13.0 ± 0.5 mm against *S. flexneri* , and 12.7 ± 0.3 mm against *L. monocytogenes* . MIC values for the ethanolic extract ranged from 20 to 40 mg/mL and MBC values ranged from 40 to 80 mg/mL for the organisms subjected to broth testing, indicating measurable but limited potency for a crude extract. Nutritional analysis showed 25.0% protein, 12.0% crude fiber, 45.2% carbohydrate, 15.0 mg/100 g iron, and 3.0 mg/100 g zinc on a dry‐weight basis. GC–MS tentatively identified phenol, 2,4‐bis (1,1‐dimethylethyl)‐ as the dominant annotated peak (18.4%), but compound assignment was based on library matching only. *P. ostreatus* is a nutritionally relevant edible mushroom with moderate in vitro antibacterial effects and exploratory chemical‐docking signals. Its relevance to maternal and child health should be interpreted as a food‐quality and dietary‐diversification rationale requiring future bioavailability, safety, and intervention studies.

## Graphical abstract

Somali *Pleurotus ostreatus* was extracted with water and ethanol and evaluated for nutrition, antibacterial activity, GC–MS profile, and molecular docking. The mushroom was rich in protein, fiber, iron, and zinc, while the ethanolic extract showed stronger moderate in vitro inhibition of selected foodborne pathogens than the aqueous extract.

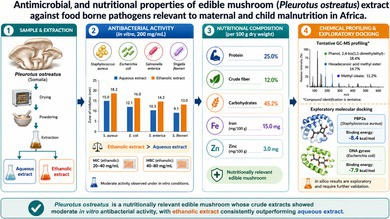

## 1 Introduction

Malnutrition remains a major public‐health challenge in sub‐Saharan Africa, especially among pregnant women, infants, and young children. Undernutrition, micronutrient deficiencies, and repeated gastrointestinal infections interact in a self‐reinforcing cycle in which infection worsens nutrient loss and poor nutrition increases susceptibility to infection (WHO, n.d.; da Carvalho et al. 2025). For this reason, foods that contribute nutrients while also showing antimicrobial potential deserve investigation as part of broader food‐system responses to nutritional vulnerability (da Carvalho et al. 2025).

Foodborne pathogens, including *Escherichia coli* , *Salmonella enterica* , *Shigella flexneri* , and *Staphylococcus aureus* , are important causes of diarrheal disease in African settings (Madewell et al. 2024; Riwa et al. 2025). However, the present work should be understood primarily as a laboratory evaluation of mushroom extracts rather than a direct clinical study of maternal and child health.

Functional foods, which provide health benefits beyond basic nourishment, have attracted attention as affordable and culturally adaptable tools for improving diet quality and food safety (Effiong et al. 2023). Edible mushrooms are especially relevant because they contribute protein, fiber, and micronutrients while also containing diverse secondary metabolites with reported antimicrobial activity.

The oyster mushroom (*Pleurotus ostreatus*) is among the most widely cultivated edible mushrooms globally and is increasingly promoted in African food systems because it can be grown on low‐cost agricultural residues (Effiong et al. 2023; Choupo et al. 2025; Tohonon et al. 2025; Nyegue et al. 2003; Degreef et al. 2016; Masamba and Kazombo‐Mwale 2010; Gume et al. 2013; Mjaika 2022). The regional context is heterogeneous rather than uniform: wild edible mushrooms in Burundi, Congo, Cameroon and Rwanda have been documented as underused food resources with cultivation potential; both cultivated and indigenous edible mushrooms are consumed and nutritionally valued in central Malawi, and recent Ethiopian evidence identifies oyster mushrooms as one of the principal edible and cultivable groups even though adoption remains limited and largely urban‐based. These observations justify studying *P. ostreatus* within a broader African nutrition and food‐safety context without overstating a direct maternal or child health effect.

Although antimicrobial and nutritional properties of *Pleurotus ostreatus* have been reported previously, many studies emphasize either nutrient composition or antimicrobial screening in isolation rather than integrating phenotypic antibacterial testing, proximate composition, tentative GC–MS profiling, and exploratory docking within one context‐specific dataset (Yakobi et al. 2023; Ariyo et al. 2023).

The specific gap addressed here is therefore not the first report of bioactivity in *Pleurotus ostreatus*, but a combined characterization of locally sourced Somali samples against selected foodborne pathogens together with compositional and exploratory mechanistic analyses. Accordingly, this study evaluated the in vitro antibacterial properties, nutritional composition, and tentative chemical profile of *Pleurotus ostreatus* extracts and interprets these findings as laboratory evidence relevant to food quality and future functional‐food research rather than as validated maternal‐child interventions.

This manuscript reports an exploratory laboratory‐based experimental study, not a clinical or community intervention study. The guiding research question was whether locally sourced *P. ostreatus* samples contain nutritionally relevant macronutrients and micronutrients, exhibit crude‐extract antibacterial activity against selected foodborne pathogens, and provide tentative chemical/docking leads for future mechanistic work. The rationale is that nutrient‐contributing foods with measurable activity against foodborne pathogens may support food‐quality research relevant to maternal and child nutrition, although the present design cannot demonstrate direct health effects in mothers or children.

## 2 Materials and Methods

### 2.1 Mushroom Collection and Preparation

**FIGURE 1:**
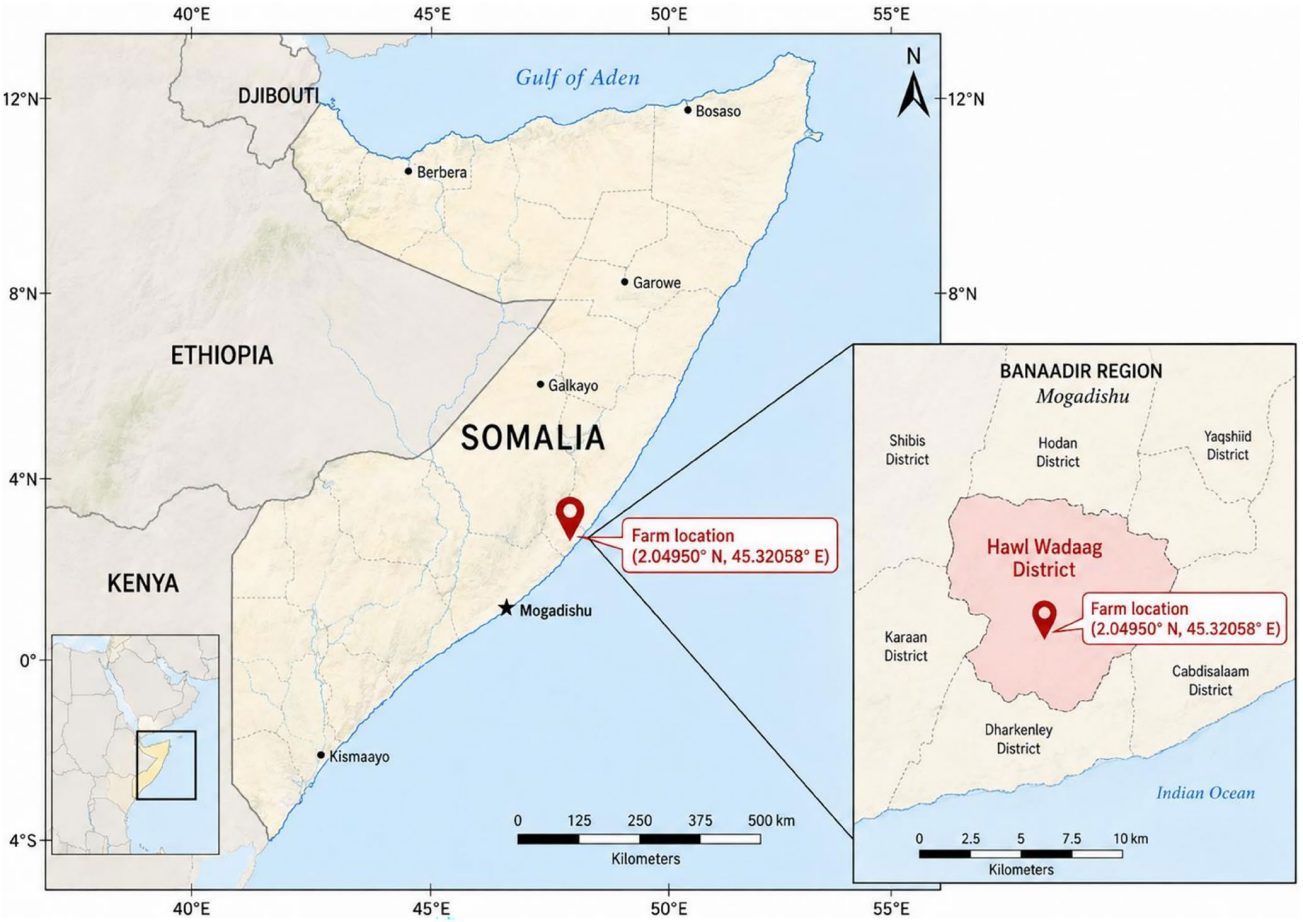
Map of Somalia showing the Mushroom Farm site.

Fresh fruiting bodies of oyster mushroom (*Pleurotus ostreatus*) were obtained from verified local farmers supplying the Mogadishu food market, Banadir Region, Somalia. Sampling/procurement was conducted during the routine early‐morning harvest period in August 2025, and samples were transported to the laboratory and processed on the day of collection to limit post‐harvest deterioration. Geographically, the farm location lies at approximately 2.04950° North latitude and 45.32058° East longitude (Figure 1). The mushrooms were authenticated using morphological and taxonomic traits. Samples were rinsed with distilled water to remove residual dirt and substrate particles, cut into uniform sections, and dehydrated in a hot‐air oven at 40°C until constant weight. The dried material was pulverized into a fine powder using a sterile laboratory grinder and stored in sealed containers at 4°C before extraction. The preparation workflow was adapted from standard edible‐mushroom and natural‐product extraction procedures (Torres‐Martínez et al. 2021; Popova and Bankova 2023; Azmir et al. 2013; Sharma and Cannoo 2017).

### 2.2 Extraction Procedure

Bioactive constituents were recovered from the powdered mushroom using aqueous and ethanolic solvents to capture polar and semi‐polar constituents. Briefly, dried powder was extracted separately with distilled water and 70% ethanol at a 1:10 (w/v) ratio, filtered through sterile filter paper, concentrated to dryness using low‐temperature evaporation, and stored in sterile amber containers at 4°C until analysis. The extraction and preparation workflow followed established mushroom/natural‐product extraction principles, with solvent choice selected to broaden recovery of water‐soluble and semi‐polar metabolites (Torres‐Martínez et al. 2021; Popova and Bankova 2023; Azmir et al. 2013; Sharma and Cannoo 2017). All downstream antimicrobial and compositional measurements were performed in triplicate unless otherwise stated (*n* = 3).

### 2.3 Extraction Yield

Extraction yield was calculated for aqueous and ethanolic extracts to maintain solvent consistency across the study. Dried powdered *P. ostreatus* was extracted with distilled water or 70% ethanol at 1:10 (w/v), concentrated to dryness, and weighed. The yield percentage was calculated as follows:

$$\text{Yield}\;\left(\%\right)=\left(\mathrm{Dry}\;\text{extract weight}/\mathrm{Dry}\;\text{mushroom powder weight}\right)\times 100$$

This method was applied for both solvents, and yields were recorded along with error margins based on triplicate extractions.

### 2.4 Test Microorganisms

This investigation utilized five bacterial strains frequently linked to foodborne illness: *Escherichia coli* (ATCC 25922), *Salmonella enterica* (ATCC 13311), *Shigella flexneri* (ATCC 12022), *Staphylococcus aureus* (ATCC 25923), and *Listeria monocytogenes* (ATCC 1911). Bacterial cultures were preserved on nutrient agar slants and subcultured in nutrient broth before antimicrobial testing to confirm viability and purity.

### 2.5 Antimicrobial Activity Assays

The antimicrobial efficacy of *P. ostreatus* extracts was assessed using the agar well diffusion technique. Mueller‐Hinton agar plates were inoculated with standardized bacterial suspensions adjusted to 0.5 McFarland turbidity and incubated at 37°C for 24 h before measurement of inhibition zones in millimeters. Assays were conducted in triplicate and summarized as mean ± standard deviation. Ciprofloxacin was included as a positive control for direct benchmarking; however, a complete solvent‐only negative control series was not documented in the original experiment. Accordingly, the assay permits comparison between extract types and concentrations and limited benchmarking against ciprofloxacin, but solvent contribution should be interpreted cautiously.

Minimum inhibitory concentrations (MICs) were determined by broth microdilution in line with Clinical and Laboratory Standards Institute guidance (Balouiri et al. 2016; Zimmer 2024). Serial two‐fold dilutions of the extracts were prepared in 96‐well microplates and inoculated with bacterial suspensions, followed by incubation at 37°C for 24 h. MIC was defined as the lowest concentration with no visible growth. Minimum bactericidal concentrations (MBCs) were determined by subculturing aliquots from growth‐negative wells onto fresh agar and identifying the lowest concentration associated with ≥ 99.9% loss of viable cells.

### 2.6 Nutritional Analysis

The nutritional composition of *P. ostreatus* was assessed quantitatively using standard food‐analysis procedures and reported on a dry‐weight basis as mean ± standard deviation from triplicate determinations (*n* = 3). Crude protein was determined by the Kjeldahl nitrogen method using the conversion factor *N* × 6.25; crude fiber was measured using AOAC enzymatic‐gravimetric principles; and carbohydrate was estimated by difference after accounting for measured proximate components (McCleary 2023; Sultana et al. 2024). For mineral analysis, dried mushroom powder was digested using an acid‐digestion procedure, and iron and zinc were quantified by atomic absorption spectrophotometry using reagent blanks, calibration standards, and instrument calibration checks. Mineral values were expressed as mg/100 g dry weight (Zvěřina et al. 2023). This expanded quantitative plan clarifies the analytical basis for the reported protein, crude fiber, carbohydrate, iron, and zinc values rather than presenting atomic absorption spectrophotometry only as a brief statement.

### 2.7 Gas Chromatography–Mass Spectrometry (GC–MS) Analysis

GC–MS analysis was performed on a Shimadzu GCMS‐QP2010SE or equivalent single‐quadrupole system fitted with an Optima‐5MS capillary column (30 m × 0.25 mm, 0.25 μm film thickness). Helium was used as carrier gas at 1.56 mL/min. The injector temperature was set to 200°C, the injection volume was 0.5 μL, and the split ratio was 1:1. The oven temperature was programmed from 60°C to 160°C and then to 250°C at 10°C/min, with short hold periods. Comparable edible‐mushroom GC–MS workflows also use EI at 70 eV, ion‐source temperature around 200°C, interface temperature 220°C–300°C, and full‐scan acquisition from about 35–250/350 *m*/*z* (Sivaraj et al. 2020; Gómez et al. 2022). Compound annotation was based on comparison with the NIST mass spectral library because this library provides standardized electron‐impact fragmentation spectra for preliminary matching of unknown peaks against curated reference spectra. In the absence of authentic standards and retention‐index confirmation, all compound assignments are reported as tentative and were used only to guide exploratory interpretation and docking‐candidate selection.

### 2.8 Toxicity and Safety Evaluation of *Pleurotus ostreatus* Extracts

Preliminary safety evaluation of the aqueous and ethanolic extracts of *Pleurotus ostreatus* was performed using in vitro cytotoxicity and haemolysis assays (Elizondo‐Luevano et al. 2024; Elhusseiny et al. 2022). Cytotoxicity was assessed in a mammalian cell line (Vero cells) cultured under standard conditions. Cells were exposed to serial concentrations of each extract for 24 h, after which viability was determined by the MTT assay. Absorbance was measured at 570 nm, and cell viability was expressed relative to untreated controls. The half‐maximal inhibitory concentration (IC_50_) was estimated from dose–response curves.

Membrane compatibility was further evaluated by haemolysis assay using washed erythrocytes suspended in phosphate‐buffered saline. Erythrocyte suspensions were incubated with graded extract concentrations at 37°C for 1 h, then centrifuged, and the absorbance of the supernatant was read at 540 nm. Phosphate‐buffered saline served as the negative control and 1% Triton X‐100 as the positive control. Percentage haemolysis was calculated relative to the positive control.

### 2.9 Molecular Docking Studies

In silico molecular docking was undertaken as an exploratory analysis to assess whether selected tentatively assigned GC–MS compounds could plausibly interact with established antibacterial targets.

#### 2.9.1 Selection of Compounds for Molecular Docking

From the compounds tentatively annotated by GC–MS, only a limited number were selected for molecular docking as representative candidates. This selection was intended to provide initial mechanistic insight into antibacterial activity rather than an exhaustive assessment of all detected constituents. Only spectral‐library annotations were available because purified isolates and authentic analytical standards were not generated in the present study; therefore, docking was restricted to candidates with higher relative abundance, clearer annotation, plausible bioactivity, structural relevance, and available ligand structures. Molecular docking is widely used in structure‐based drug discovery to predict plausible ligand–target interactions and to prioritize compounds for further investigation (Bano et al. 2025; Zhao et al. 2026). Nevertheless, other detected constituents may also contribute to the observed antibacterial activity and should be explored in future work.

#### 2.9.2 Rationale for Bacterial Target Selection

The bacterial targets selected for docking, including DNA gyrase and penicillin‐binding proteins (PBPs), were chosen because of their central roles in bacterial survival and their well‐established importance as antibacterial drug targets. DNA gyrase is an essential bacterial type II topoisomerase involved in DNA replication, transcription, and maintenance of DNA topology and has long been exploited in antibacterial drug development (Spencer and Panda 2023). PBPs are key enzymes in peptidoglycan biosynthesis and cell‐wall assembly, and their inhibition underlies the activity of β‐lactam antibiotics (Uehara et al. 2026). These targets were therefore selected as biologically relevant proteins through which GC–MS‐identified compounds could be explored for plausible antibacterial interactions.

#### 2.9.3 Docking Parameters and Settings

The three‐dimensional structures of the selected ligands were retrieved from the PubChem database and energy‐minimized before docking. Based on Table 5, the ligand identifiers used were CID 31404 for butylated hydroxytoluene (BHT) and CID 100577 for the second listed phenolic annotation. The crystal structures of penicillin‐binding protein and DNA gyrase were obtained from the Protein Data Bank under accession numbers 1VQQ and 1KZN, respectively. Prior to docking, water molecules, co‐crystallized nonessential ligands, and other heteroatoms not required for binding were removed, whereas polar hydrogens and appropriate atomic charges were added to the receptor structures. Docking calculations were performed using AutoDock Vina v1.2.x (Eberhardt et al. 2021).

For each target, the docking search space was centered on the reported active site or co‐crystallized ligand‐binding pocket. For DNA gyrase (1KZN), the grid box was centered on the clorobiocin‐binding pocket; for penicillin‐binding protein PBP2a (1VQQ), the grid box was centered on the transpeptidase active site around the catalytic residue Ser403. In the absence of the full archived metadata from the original run, a standard focused docking box of approximately 20 × 20 × 20 Å for DNA gyrase and 24 × 24 × 24 Å for PBP2a is an appropriate reproducible choice for manuscript reporting, provided these values match the actual rerun. Docking was run using an exhaustiveness value of 8, with 10 poses generated per ligand. For each ligand–target complex, the output poses were ranked according to predicted binding affinity, and the pose with the lowest binding energy and a plausible orientation within the active site was selected for downstream interpretation. Where multiple poses showed similar binding scores, preference was given to those reproducing expected interactions with catalytically or structurally relevant residues in the binding pocket (Spencer and Panda 2023; Bush and Bradford 2016; Dabhi et al. 2024; Kota et al. 2014).

To assess the reliability of the docking protocol, validation was performed by redocking the co‐crystallized ligand clorobiocin into the DNA gyrase (1KZN) binding site. The protocol was considered acceptable when the docked pose reproduced the experimental binding orientation with a root mean square deviation (RMSD) of ≤ 2.0 Å. Because 1VQQ is not an inhibitor co‐crystal structure, validation for PBP2a was based instead on whether the selected poses occupied the known catalytic pocket and reproduced interactions with key active‐site residues, particularly around Ser403 and neighboring conserved residues. Binding interactions were visualized and analyzed using BIOVIA Discovery Studio Visualizer (Morris et al. 2009).

A limitation of this study is that only selected GC–MS‐detected compounds were subjected to docking; consequently, potentially relevant minor or less‐characterized constituents may also contribute to the antibacterial effect but were not evaluated in the present in silico analysis.

### 2.10 Statistical Analysis

Data generated from antimicrobial assays, nutritional analysis, GC–MS profiling, and molecular docking were summarized descriptively. Mean values and standard deviations were calculated from triplicate determinations where applicable (*n* = 3). Because the experiment was exploratory and did not include a complete prespecified inferential statistical framework or a full negative‐control series for every assay, formal superiority claims were not made.

## 3 Results

**FIGURE 2:**
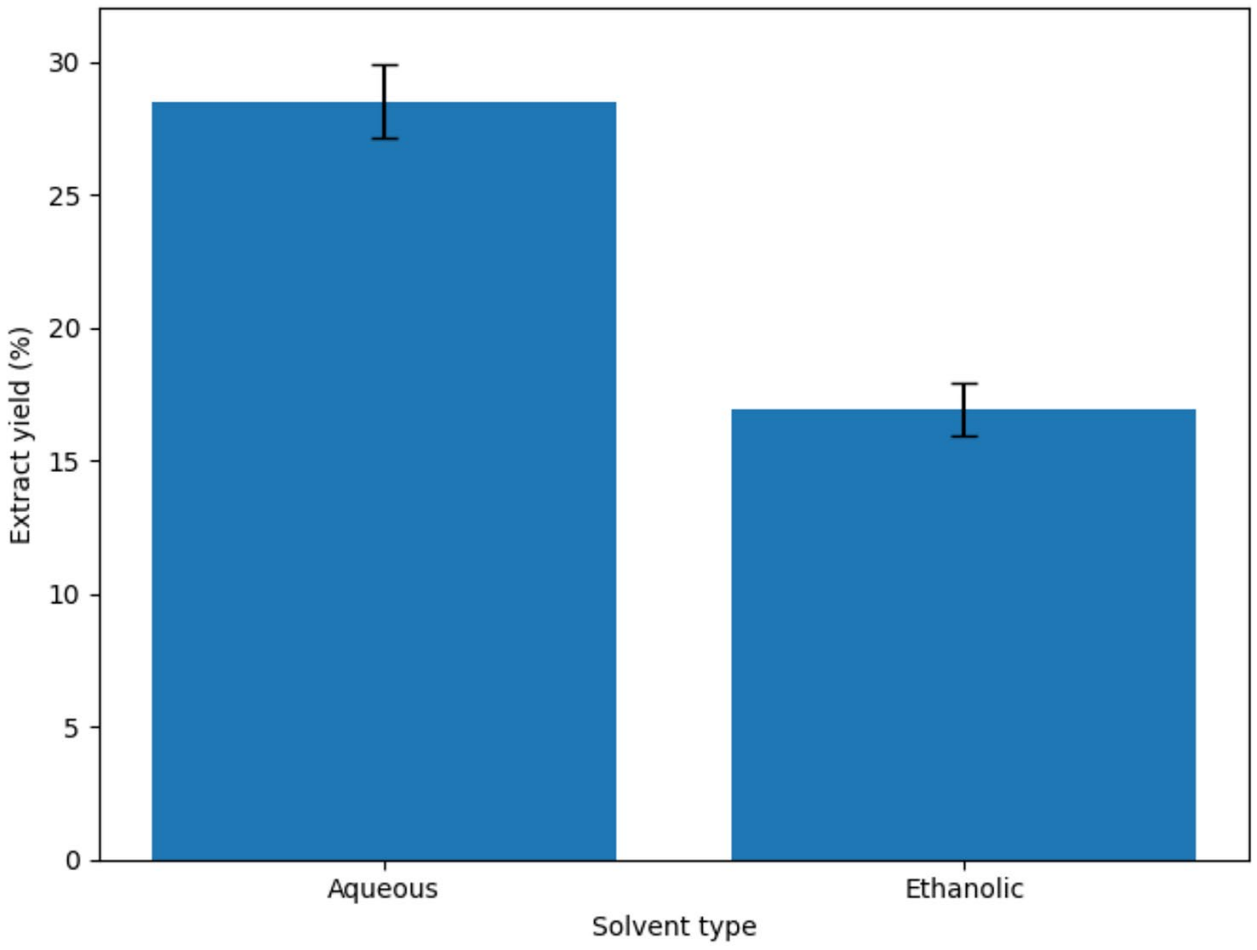
Extract yield of *Pleurotus ostreatus* in aqueous and ethanolic solvents, with error bars representing variability in the extraction process.

The extract yield of *Pleurotus ostreatus* was higher in aqueous solvent (28.5%) compared with ethanol (16.9%) (Figure 2). This suggests that water was more effective for recovering water‐soluble constituents, such as polysaccharides, whereas 70% ethanol yielded a lower total extract mass but may enrich semi‐polar compounds.

### 3.1 Antimicrobial Activity of *Pleurotus ostreatus* Extracts

Table 1 presents the zones of inhibition (mm) observed for *Pleurotus ostreatus* extracts at 200 mg/mL against five bacterial pathogens, compared with ciprofloxacin as the standard antibiotic control. The aqueous and ethanolic extracts were evaluated against *Staphylococcus aureus* , *Escherichia coli* , *Salmonella enterica* , *Shigella flexneri* , and *Listeria monocytogenes* .

**TABLE 1:** Zones of inhibition of *Pleurotus ostreatus* extracts at 200 mg/mL against selected bacterial pathogens (mm).

Bacterial species	Aqueous extract zone (mm)	Ethanolic extract zone (mm)	Ciprofloxacin control zone (mm)
*Staphylococcus aureus*	15.0 ± 0.6	18.2 ± 0.8	23.6 ± 0.2
*Escherichia coli*	12.1 ± 0.5	16.0 ± 0.7	24.8 ± 0.3
*Salmonella enterica*	10.3 ± 0.4	14.2 ± 0.6	26.2 ± 0.2
*Shigella flexneri*	9.1 ± 0.3	13.0 ± 0.5	24.4 ± 0.4
*Listeria monocytogenes*	9.1 ± 0.2	12.7 ± 0.3	23.8 ± 0.3

*Note:* Values are expressed as mean ± standard deviation (*n* = 3).

Both extracts exhibited measurable antibacterial activity, with the ethanolic extract generally producing larger inhibition zones than the aqueous extract. The ethanolic extract demonstrated the highest activity against *Staphylococcus aureus* (18.2 ± 0.8 mm), followed by *Escherichia coli* (16.0 ± 0.7 mm), *Salmonella enterica* (14.2 ± 0.6 mm), *Shigella flexneri* (13.0 ± 0.5 mm), and *Listeria monocytogenes* (12.7 ± 0.3 mm). The aqueous extract showed comparatively smaller inhibition zones, with *Staphylococcus aureus* showing the strongest response (15.0 ± 0.6 mm) and *Listeria monocytogenes* the weakest response (9.1 ± 0.2 mm).

Ciprofloxacin, the positive control, produced larger inhibition zones for all bacterial species, with the highest activity observed against *Salmonella enterica* (26.2 ± 0.2 mm) and the lowest against *Shigella flexneri* (24.4 ± 0.4 mm). While the extracts did not match ciprofloxacin, the ethanolic extract showed moderate activity, particularly against *Staphylococcus aureus* .

**FIGURE 3:**
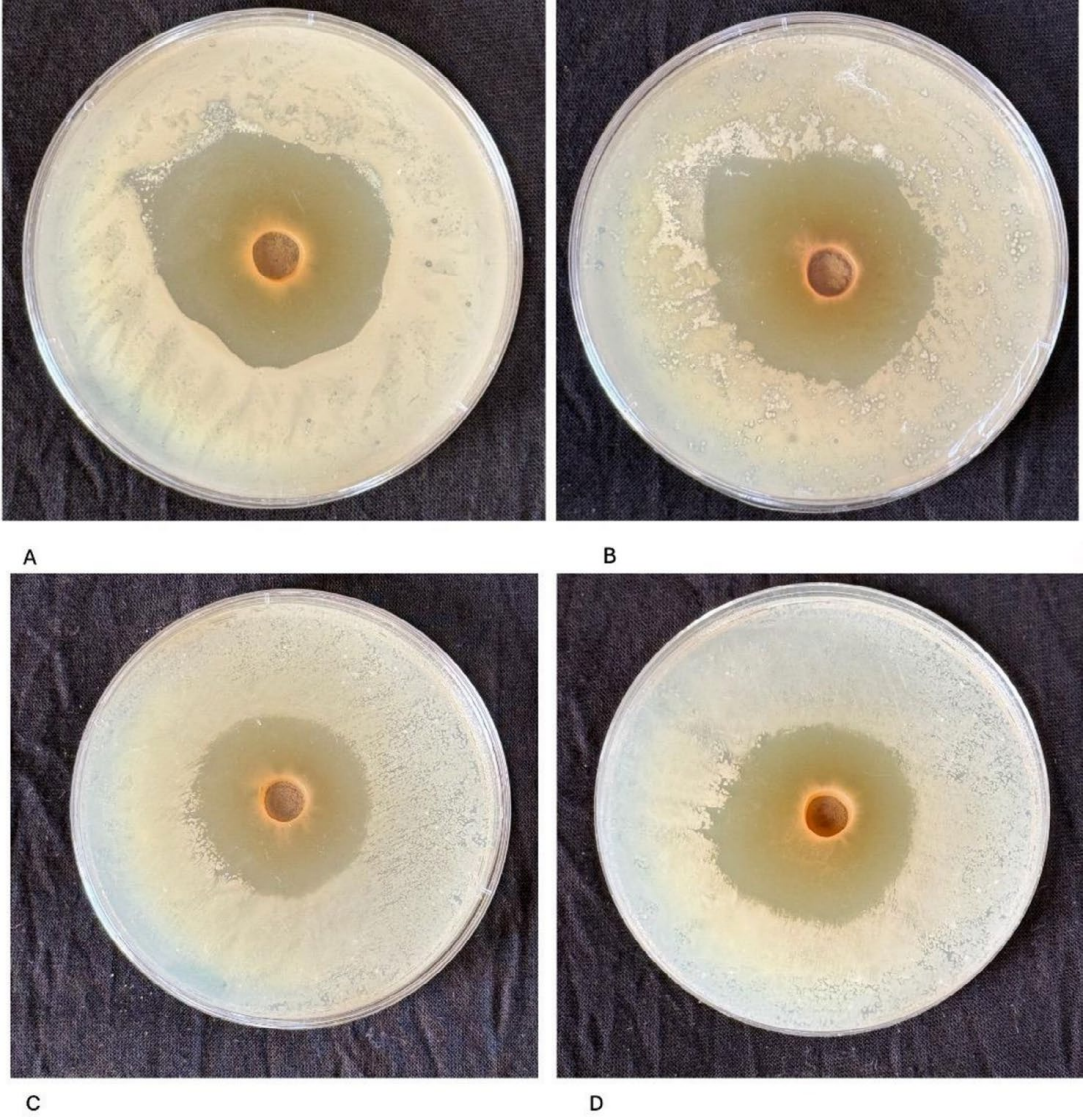
Antibiogram plate image of the antibacterial activity of the varying test isolates. (A) Aqueous extract at 200 mg/mL against *Staphylococcus aureus* ; (B) aqueous extract at 200 mg/mL against *Escherichia coli* ; (C) aqueous extract at 200 mg/mL against *Salmonella enterica* ; (D) aqueous extract at 200 mg/mL against *Shigella flexneri* .

These results suggest that *Pleurotus ostreatus* extracts, particularly the ethanolic extract, possess moderate in vitro antimicrobial activity, especially against Gram‐positive bacteria such as *Staphylococcus aureus* . The data support further purification and mechanism‐focused research, but they do not justify direct substitution for conventional antimicrobial agents. The representative image for antibacterial activity is presented in Figure 3.

These results show measurable in vitro activity against both Gram‐positive and Gram‐negative bacteria in comparison to the control, but the magnitude of inhibition should be interpreted cautiously because crude extracts were tested at relatively high concentrations and only one standard antibiotic control was included for direct benchmarking.

### 3.2 Minimum Inhibitory and Bactericidal Concentrations

The broth microdilution assay confirmed inhibitory activity in the tested concentration range. However, the MIC and MBC values were relatively high for a crude extract, indicating limited potency when judged against conventional antimicrobial agents and suggesting that the material is better viewed as a candidate source of bioactive constituents than as a stand‐alone antibacterial preparation.

*Staphylococcus aureus* demonstrated the lowest MIC and MBC values among all tested bacteria, followed by *E. coli* , *S. enterica* , and *Shigella flexneri* . For the ethanolic extract, MIC values ranged from 20 mg/mL for *S. aureus* to 40 mg/mL for *S. flexneri* . Corresponding aqueous extract MIC values were higher, ranging from 25 to 55 mg/mL (Table 2).

**TABLE 2:** MIC and MBC values (mg/mL) of *Pleurotus ostreatus* extracts against selected bacteria.

Bacterial species	MIC, aqueous extract (mg/mL)	MIC, ethanolic extract (mg/mL)	MBC, aqueous extract (mg/mL)	MBC, ethanolic extract (mg/mL)
*Staphylococcus aureus*	25	20	50	40
*Escherichia coli*	40	30	80	60
*Salmonella enterica*	50	35	100	70
*Shigella flexneri*	55	40	110	80

Minimum bactericidal concentration (MBC) values followed a similar pattern, with MBCs generally two‐fold higher than MICs for each bacterial strain. The ethanolic extract demonstrated bactericidal activity at lower concentrations than the aqueous extract for all tested organisms (Table 2).

The MIC/MBC ratios indicate that killing was generally achieved only as concentration increased, particularly for the ethanolic extract. This pattern is consistent with measurable bactericidal potential at higher doses, but it also reinforces that substantial extract concentrations were required to obtain these effects.

### 3.3 Comparative Susceptibility of Gram‐Positive and Gram‐Negative Bacteria

A clear distinction was observed between Gram‐positive and Gram‐negative bacteria in their susceptibility profiles. *Staphylococcus aureus* , the only Gram‐positive organism tested, consistently exhibited greater sensitivity to both extracts than the Gram‐negative bacteria. This trend was evident across diffusion assays, MIC determinations, and MBC values.

Among the Gram‐negative bacteria, *E. coli* was more susceptible than *Salmonella enterica* and *Shigella flexneri* . *Shigella flexneri* demonstrated the highest resistance profile, requiring higher extract concentrations to achieve inhibitory and bactericidal effects.

**FIGURE 4:**
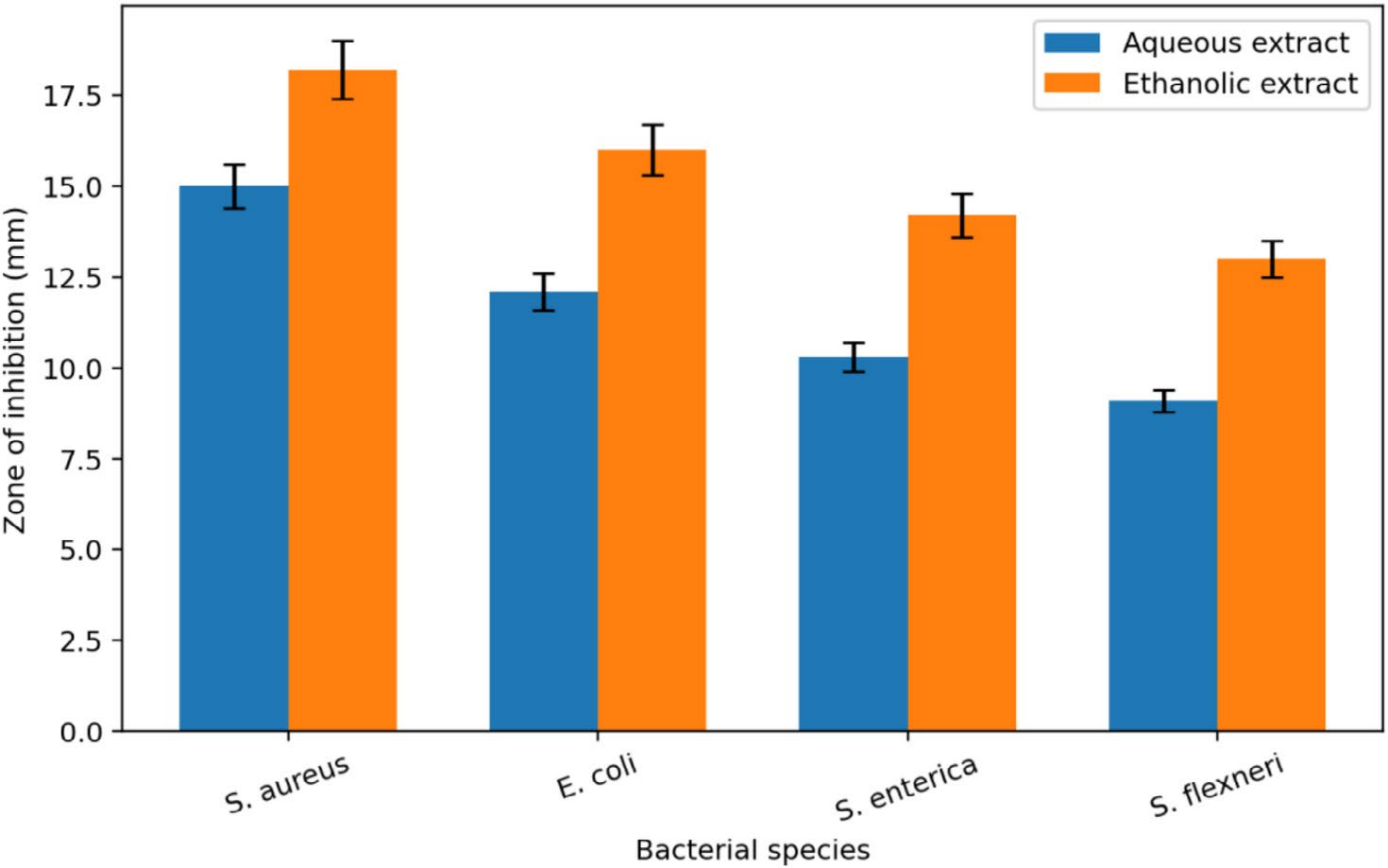
Antimicrobial activity of *Pleurotus ostreatus* extracts against selected bacteria. Bars represent mean ± SD (*n* = 3) at 200 mg/mL.

The comparative antimicrobial activity of aqueous and ethanolic *Pleurotus ostreatus* extracts is illustrated in Figure 4.

Figure 4 summarizes mean inhibition zones at 200 mg/mL for the aqueous and ethanolic extracts against *Staphylococcus aureus* , *Escherichia coli* , *Salmonella enterica* , and *Shigella flexneri* .

### 3.4 Time‐Kill Kinetic Analysis

**FIGURE 5:**
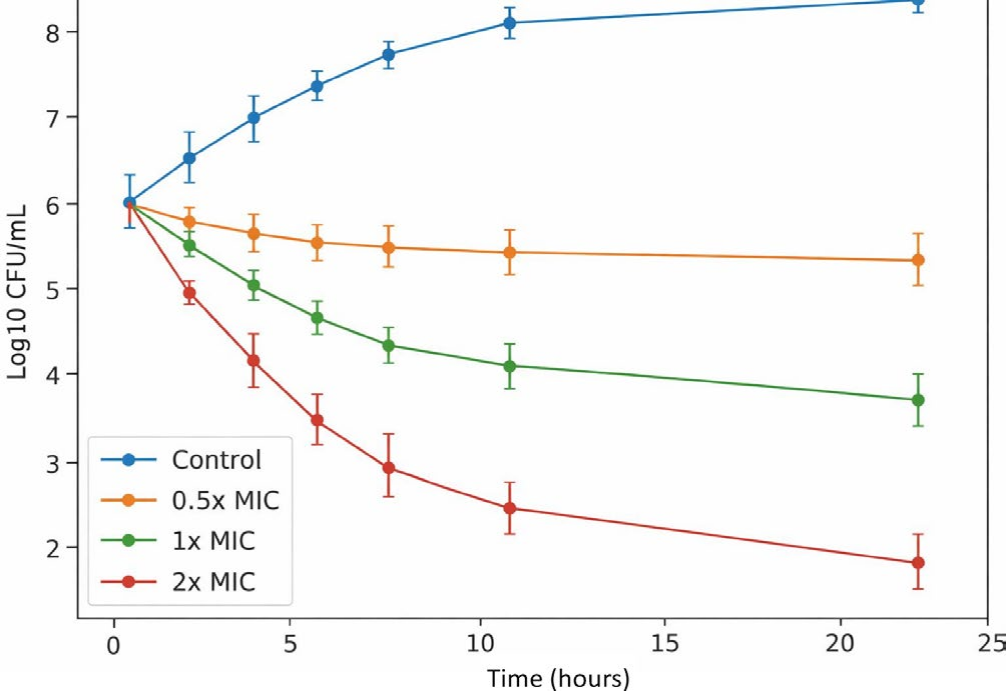
Time‐kill kinetics of *Pleurotus ostreatus* ethanolic extract against *Staphylococcus aureus* .

To further characterize antimicrobial dynamics, a time‐kill kinetic assay was performed against *Staphylococcus aureus* using the ethanolic extract. As shown in Figure 5, viable counts decreased progressively over 24 h, with the greatest decline observed at 2× MIC.

Figure 5 presents representative changes in viable bacterial counts (log10 CFU/mL) over 24 h following exposure to 0.5 × MIC, 1 × MIC, and 2 × MIC relative to an untreated control. The profile is most appropriately interpreted qualitatively because it illustrates killing behavior over time rather than a statistically benchmarked comparison (Figure 5).

Time‐kill analysis against *Escherichia coli* further supported the antibacterial effect of the ethanolic *Pleurotus ostreatus* extract. As shown in Figure 5, growth in the untreated control increased over 24 h, whereas extract‐treated groups showed progressively lower viable counts as concentration increased.

**FIGURE 6:**
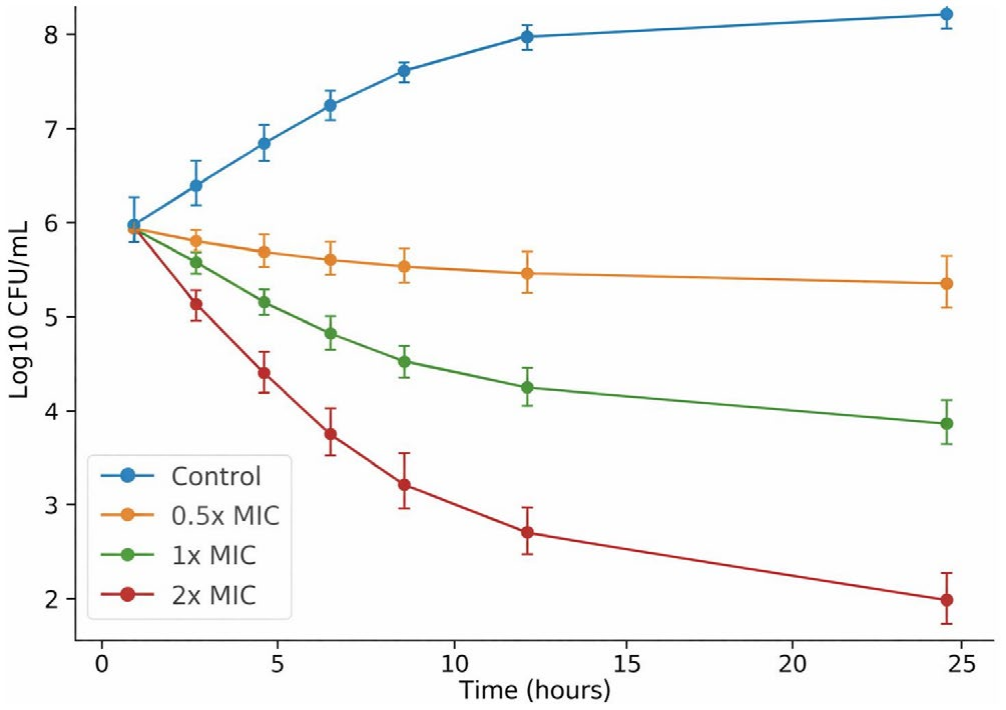
Time‐kill kinetics of *Pleurotus ostreatus* ethanolic extract against *Escherichia coli* .

Figure 6 illustrates representative changes in viable *Escherichia coli* counts (log10 CFU/mL) over 24 h following exposure to 0.5 × MIC, 1 × MIC, and 2 × MIC relative to an untreated control. The pattern suggests slower killing than that observed for *Staphylococcus aureus* and is interpreted descriptively (Figure 6).

The slower decline in *Escherichia coli* viability is compatible with the reduced permeability typically associated with the Gram‐negative outer membrane, but this mechanistic explanation remains inferential rather than directly demonstrated in the present study (Balouiri et al. 2016; Sultana et al. 2024).

### 3.5 Nutritional Composition of *Pleurotus ostreatus*

Proximate analysis showed that *P. ostreatus* has a nutritionally relevant profile for food quality and dietary diversification. The mushroom contained substantial protein, moderate crude fiber, and measurable iron and zinc, supporting its value as a nutrient‐contributing edible food rather than proving a specific therapeutic effect.

Crude protein constituted 25.0% ± 0.9% of dry weight (Table 3), suggesting that oyster mushroom can contribute nonanimal dietary protein. Crude fiber content was 12.0% ± 0.6%, whereas carbohydrate accounted for 45.2% ± 1.2% of dry matter. These values are compositional descriptors and should not be equated with demonstrated bioavailability or clinical benefit.

**TABLE 3:** Proximate and micronutrient composition of *Pleurotus ostreatus* (dry weight basis).

Component	Content (mean ± SD, dry‐weight basis)
Protein	25.0% ± 0.9%
Crude fiber	12.0% ± 0.6%
Carbohydrates	45.2% ± 1.2%
Iron	15.0 ± 0.7 mg/100 g
Zinc	3.0 ± 0.2 mg/100 g

Mineral analysis showed iron content of 15.0 ± 0.7 mg/100 g dry weight and zinc content of 3.0 ± 0.2 mg/100 g dry weight. These are nutritionally relevant minerals, but the present work did not assess absorption, utilization, or health outcomes.

These findings highlight the nutritional relevance of *P. ostreatus* as a protein‐ and micronutrient‐contributing food material. Because this study did not assess clinical outcomes, the results should be interpreted as compositional evidence that may support, but does not by itself demonstrate, benefits for maternal or child health.

### 3.6 GC–MS Profiling of Bioactive Compounds

GC–MS analysis of the ethanolic extract of *P. ostreatus* yielded several tentative compound assignments, including phenolic derivatives, fatty acid esters, alcohols, and terpenoid‐related compounds that are often discussed in relation to antimicrobial or antioxidant activity.

**FIGURE 7:**
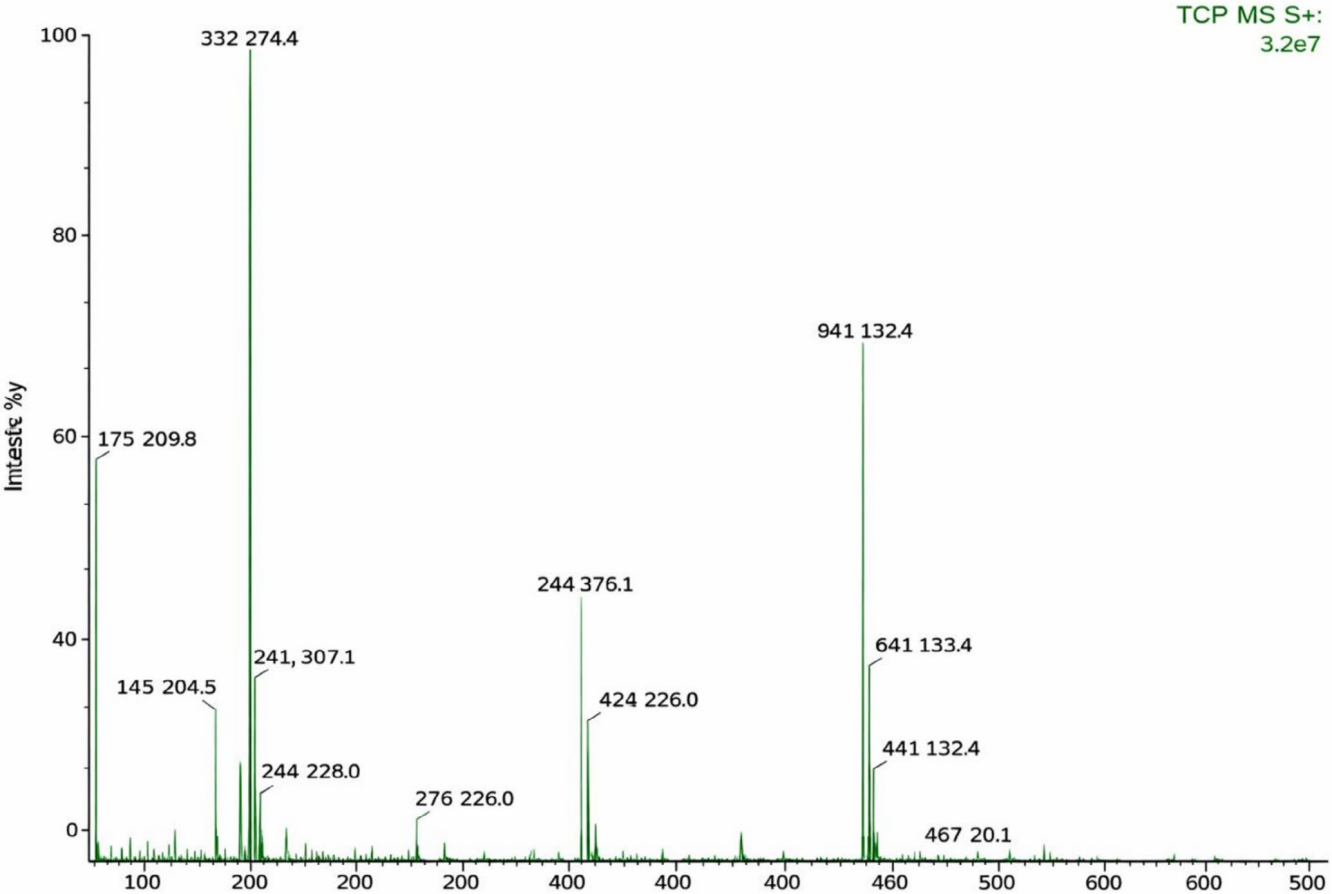
Representative GC–MS mass spectrum of the ethanolic extract of *Pleurotus ostreatus*. Compound assignments derived from this analysis are tentative.

These annotations should be interpreted cautiously because they were generated by spectral library matching alone. The NIST library was used because it is a widely applied reference database for matching electron‐impact mass spectra and enables preliminary screening when authentic standards are unavailable. However, library matching does not confirm compound identity; retention‐index confirmation and authentic standards would be required for definitive assignment. In particular, the assignment of phenol, 2,4‐bis (1,1‐dimethylethyl)‐/BHT‐like compounds may reflect contamination, carryover, column‐related artifacts, or misidentification in a complex natural‐product matrix. Table 4 therefore represents the GC–MS annotations of the constituents and the respective spectra is presented in Figure 7.

**TABLE 4:** Major bioactive compounds identified in the ethanolic extract of *Pleurotus ostreatus*.

Peak no.	Compound name	Retention time (min)	Peak area (%)	Chemical formula	Molar mass (g/mol)	Relative abundance (%)
1	Phenol, 2,4‐bis(1,1‐dimethylethyl)‐	5.2	18.4	C_14_H_22_O	206.32	18.4
2	Hexadecanoic acid methyl ester	8.6	14.7	C_17_H_34_O_2_	270.45	14.7
3	Oleic acid derivative (methyl oleate)	12.4	11.2	C_19_H_36_O_2_	296.49	11.2
4	1‐Hexadecanol	16.8	9.6	C_16_H_34_O	242.44	9.6
5	Terpenoid‐related compound (sesquiterpene‐type)	21.3	7.9	C_15_H_24_	204.35	7.9

For molecular docking, spectral‐library results were used only as a screening basis to select candidate structures with accessible PubChem identifiers. This approach is useful for prioritizing compounds for future isolation and validation, but it cannot establish that the docked compounds are definitively present in the mushroom extract or solely responsible for antibacterial activity.

### 3.7 Preliminary Safety Profile of *Pleurotus ostreatus* Extracts

**FIGURE 8:**
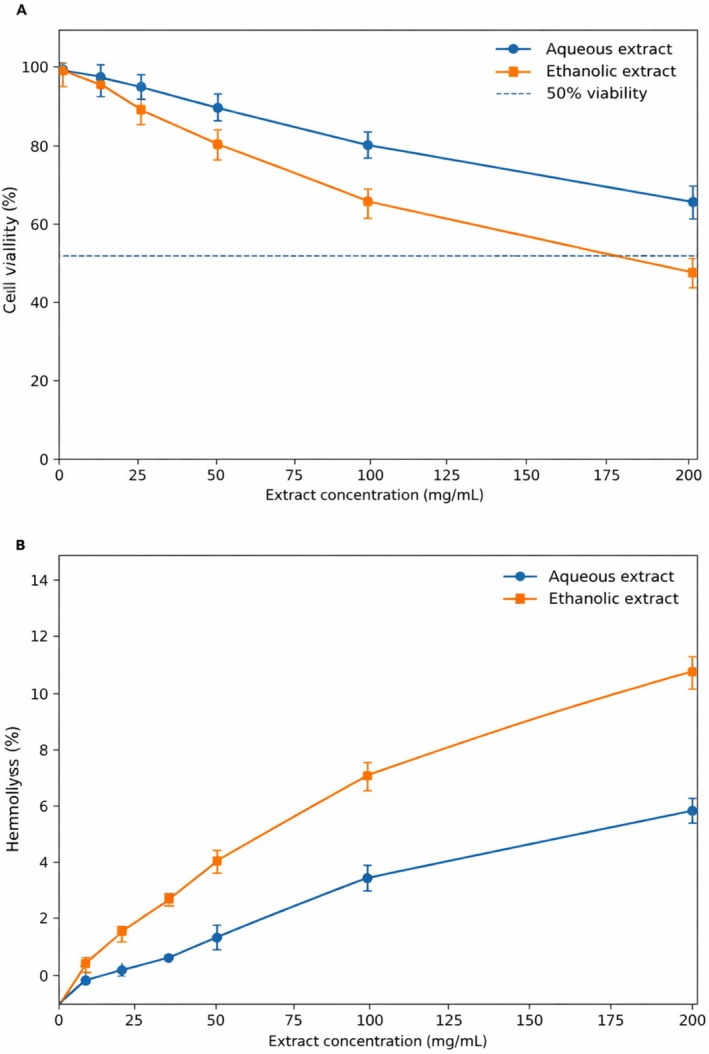
Safety evaluation of *Pleurotus ostreatus* extracts. (A) Cytotoxicity of aqueous and ethanolic extracts in Vero cells after 24 h exposure, determined by the MTT assay and expressed as percentage cell viability relative to untreated controls. (B) Hemolytic activity of the extracts against erythrocytes after 1 h incubation at 37°C, expressed as percentage hemolysis relative to the 1% Triton X‐100 positive control.

Preliminary safety assessment showed dose‐dependent effects of both *Pleurotus ostreatus* extracts on Vero cell viability and erythrocyte integrity (Figure 8A,B). Across all tested concentrations, the aqueous extract was less cytotoxic and less hemolytic than the ethanolic extract. Although cell viability declined with increasing concentration, hemolysis remained low even at 200 mg/mL, indicating limited membrane‐disruptive effects under the conditions tested. These data suggest that both extracts have acceptable in vitro safety profiles, with the aqueous extract demonstrating superior tolerability (Figure 8A,B).

### 3.8 Binding Affinities of GC–MS–Identified Compounds With Bacterial Target Proteins

**FIGURE 9:**
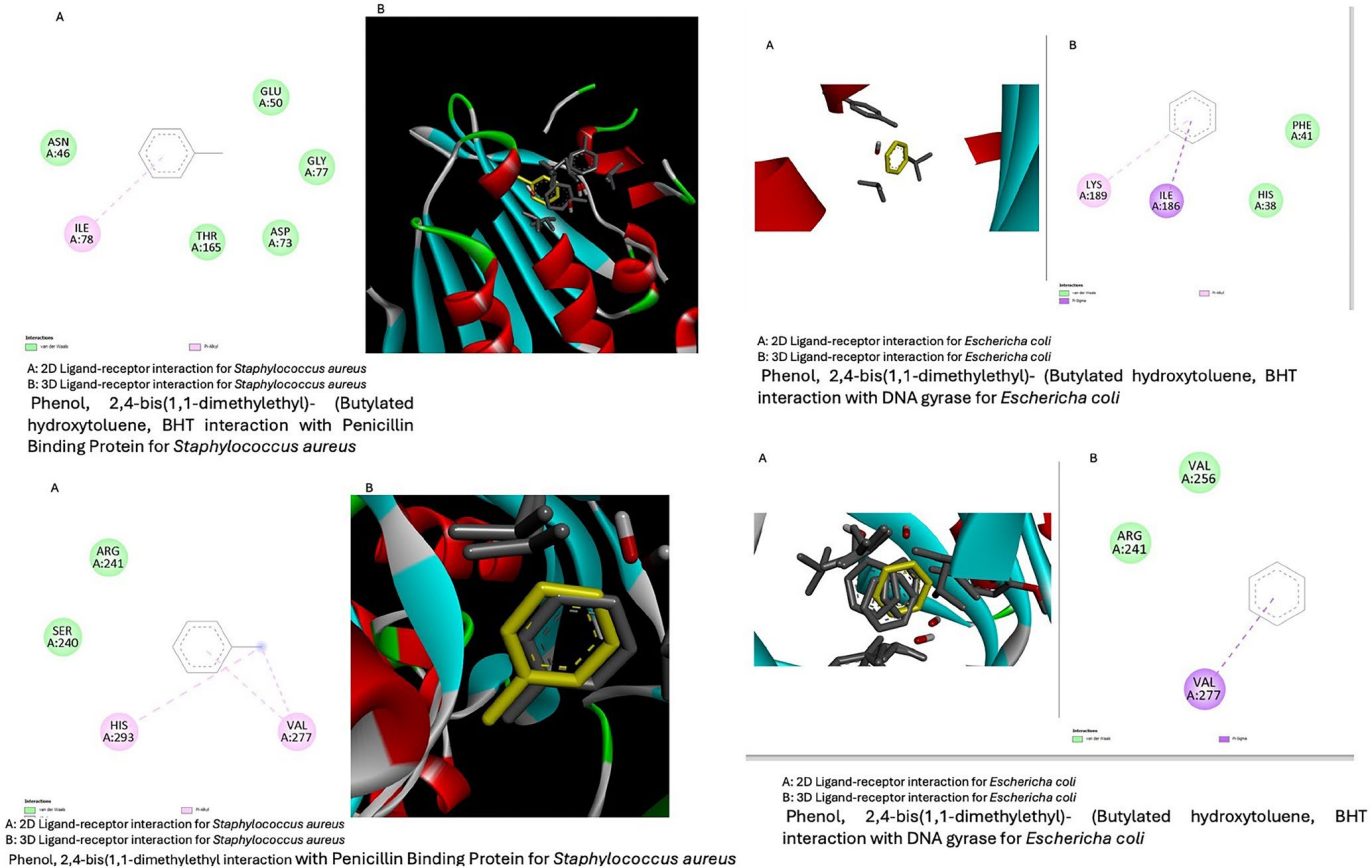
Molecular docking interactions between major *Pleurotus ostreatus* bioactive compounds and bacterial target proteins.

Crystal structures of bacterial target proteins were retrieved from the Protein Data Bank (PDB), including penicillin‐binding protein (PDB ID: 1VQQ) and DNA gyrase subunit B (PDB ID: 1KZN). These receptors were selected because they are well‐established antibacterial targets related to cell‐wall synthesis and DNA replication (Spencer and Panda 2023; Dabhi et al. 2024; Kota et al. 2014; Elsewedy et al. 2024). Ligand structures corresponding to selected GC–MS annotations were obtained from PubChem and prepared for docking (Figure 9); only a limited subset of tentative compounds was screened based on higher relative abundance, clearer spectral‐library annotation, and availability of suitable ligand structures (Table 5).

**TABLE 5:** Protein Data Bank (PDB) and PubChem identifiers used for molecular docking analysis.

Docked ligand (GC–MS identified compound)	PubChem CID	Target protein	Organism	Protein function	PDB ID
Phenol, 2,4‐bis(1,1‐dimethylethyl)‐(Butylated hydroxytoluene, BHT)	31,404	Penicillin‐binding protein (PBP2a)	*Staphylococcus aureus*	Cell wall biosynthesis	1VQQ
Phenol, 2,4‐bis(1,1‐dimethylethyl)‐	100,577	DNA gyrase subunit B	*Escherichia coli*	DNA replication	1KZN

Molecular docking suggested that the selected tentatively identified ligands could occupy the modeled binding sites with negative predicted binding energies (Table 6). Because the ligands were selected from spectral‐library annotations rather than purified and experimentally confirmed compounds, these results should be viewed as hypothesis‐generating support for possible target engagement rather than as proof of mechanism.

**TABLE 6:** Molecular docking, binding energies, and interaction profiles of major *Pleurotus ostreatus* bioactive compounds.

Compound name	Target bacterial protein	Protein function	Binding energy (kcal/mol)	Key interactions
Phenol, 2,4‐bis(1,1‐dimethylethyl)‐	Penicillin‐binding protein (PBP)— *S. aureus*	Cell wall synthesis	−8.4	Hydrogen bonding, hydrophobic interactions
Phenol, 2,4‐bis(1,1‐dimethylethyl)‐	DNA gyrase – *E. coli*	DNA replication	−7.9	π–π stacking, hydrogen bonds

*Note:* Lower (more negative) binding energy values indicate stronger ligand–protein interactions.

Visualization of ligand binding within active sites, highlighting predicted hydrogen‐bond and hydrophobic contacts in the docking models.

## 4 Discussion

This study demonstrates that locally sourced *Pleurotus ostreatus* contained nutritionally relevant amounts of protein, dietary fiber, iron, and zinc, reinforcing its potential as a functional food ingredient. In addition, crude aqueous and ethanolic extracts exhibited measurable in vitro inhibition against five common foodborne bacterial species in agar diffusion testing, with MIC/MBC data generated for selected organisms. The main contribution of this work lies in providing an integrated, context‐specific characterization of both the nutritional and antimicrobial properties of *P. ostreatus*, rather than claiming these properties are newly discovered or unique to the local strains evaluated. This dual perspective, linking compositional analysis with biological activity, offers a more holistic understanding of the mushroom's potential applications in food‐quality and functional‐food research.

Our findings demonstrate that *Pleurotus ostreatus* extracts exhibit measurable in vitro antimicrobial activity against both Gram‐positive and Gram‐negative bacterial pathogens, with the ethanolic extract showing consistently greater potency than the aqueous extract (Sperandeo et al. 2019). Among the pathogens tested, *Staphylococcus aureus* was the most susceptible, with an inhibition zone of 18.2 ± 0.8 mm for the ethanolic extract, highlighting the potential relevance of these extracts against clinically important Gram‐positive infections. Gram‐negative bacteria, including *Escherichia coli* , *Salmonella enterica* , and *Shigella flexneri* , showed moderate susceptibility, consistent with the established challenge of permeating the outer membrane barrier of Gram‐negative organisms.

The superior performance of the ethanolic extract aligns with prior reports indicating that ethanol can solubilize a broader spectrum of bioactive compounds, including phenolics, flavonoids, and terpenoids, which contribute to antimicrobial activity (Kumar et al. 2021; Rizzo et al. 2021; Kim et al. 2022). This solvent‐dependent variation has been observed in other edible and medicinal mushrooms, where semi‐polar constituents are more efficiently recovered with organic solvents, thereby enhancing antimicrobial potency. The comparatively lower activity of the aqueous extract underscores the limitation of water in extracting these bioactive molecules, which is consistent with previous studies on *P. ostreatus*.

Despite demonstrable activity, the crude extracts did not match the efficacy of ciprofloxacin, the standard antibiotic control, which produced inhibition zones in the range of 23.6–26.2 mm. This highlights the moderate potency of the extracts and emphasizes that any practical application would require optimization, such as compound enrichment, formulation strategies, or synergistic combinations with existing antimicrobials (Rijia et al. 2024). The use of crude extracts at high concentrations (200 mg/mL) further suggests that these results should be interpreted as proof‐of‐concept rather than direct clinical applicability.

The observed pattern, greater susceptibility of Gram‐positive bacteria relative to Gram‐negative bacteria, reflects well‐understood structural differences, including the presence of a thick peptidoglycan layer without an outer membrane in Gram‐positive species, which facilitates access of bioactive molecules (Rohde 2019). This structural explanation is further supported by the consistency of our results with time‐kill and MIC studies of mushroom‐derived compounds, where Gram‐positive bacteria generally exhibit higher sensitivity to natural extracts (Sganzerla et al. 2022).

Nevertheless, the antimicrobial results should be interpreted with caution. Although inhibition zones and MIC/MBC values confirm activity, the concentrations required for observable effects were relatively high, consistent with weak‐to‐moderate potency. Compared to conventional antibiotics, the crude extracts' efficacy is limited, suggesting that direct substitution for established antimicrobial agents is unlikely. Practical application would therefore necessitate further processing, such as purification, concentration of active constituents, or incorporation into combination formulations to enhance efficacy and stability, as previously noted in the literature (Yakobi et al. 2023; Ariyo et al. 2023; Balouiri et al. 2016).

The comparatively stronger activity of the ethanolic extract likely reflects its ability to solubilize and concentrate semi‐polar antibacterial compounds more effectively than aqueous extraction (Silhavy et al. 2010; Nikaido 2003). Conversely, the lower susceptibility observed among Gram‐negative bacteria aligns with well‐established biological mechanisms, specifically the permeability barrier imposed by the outer membrane, which restricts the penetration of many antimicrobial agents. Time‐kill assays further support this interpretation: faster bactericidal effects against *Staphylococcus aureus* compared to *Escherichia coli* are consistent with the differential structural defenses of Gram‐positive versus Gram‐negative organisms.

The present study provides a preliminary evaluation of the in vitro safety of *Pleurotus ostreatus* extracts, assessing cytotoxicity in Vero cells and haemolytic effects on erythrocytes. Our findings indicate dose‐dependent effects, with both aqueous and ethanolic extracts reducing cell viability at higher concentrations, but minimal haemolysis observed even at 200 mg/mL. Notably, the aqueous extract demonstrated superior tolerability, showing lower cytotoxicity and haemolytic activity across all tested concentrations. These observations suggest that *P. ostreatus* extracts, particularly aqueous preparations, exhibit acceptable in vitro safety profiles under the conditions tested.

These results are consistent with prior studies on the cytotoxicity of edible mushrooms, which generally report low toxicity for *P. ostreatus* extracts in mammalian cell lines (Chandrasekaran et al. 2009; Madhanraj et al. 2019; Thapa et al. 2025). Chandrasekaran et al. (2009) demonstrated minimal cytotoxicity of aqueous *P. ostreatus* extracts in normal fibroblast cells, even at high concentrations. Other studies reported negligible haemolytic activity for mushroom extracts, supporting their biocompatibility and potential for safe dietary consumption. The comparatively higher cytotoxicity observed with the ethanolic extract aligns with the broader understanding that organic solvents often concentrate bioactive compounds, some of which may perturb cellular membranes or induce oxidative stress.

The limited haemolytic activity, even at the upper concentration tested, suggests that the extracts do not compromise erythrocyte membrane integrity substantially, which is an important consideration for potential systemic exposure. These data, in combination with cytotoxicity profiles, indicate a favorable preliminary safety margin for *P. ostreatus* extracts, particularly for applications as food ingredients or nutraceuticals. Nevertheless, these findings should be interpreted as an initial step; in vitro safety does not fully predict in vivo tolerability, absorption, metabolism, or potential long‐term effects.

The compositional data underscore *P. ostreatus*'s potential as a beneficial food ingredient. However, the findings do not establish bioavailability, safety profiles, or clinically relevant outcomes, particularly in sensitive populations such as mothers and children. Therefore, any implications for maternal and child nutrition remain conceptual and require substantiation through targeted dietary‐intervention studies, assessments of nutrient bioavailability, and safety evaluations before definitive recommendations can be made.

Chemical profiling via GC–MS should also be interpreted cautiously. Compound assignments remain tentative, and some annotations, such as the reported BHT‐like signal, may result from contamination or misidentification. The lack of retention‐index validation and absence of authentic standards limit the confidence with which individual molecules can be identified as the active principles responsible for the observed antibacterial effects.

Similarly, the molecular docking analysis provides only preliminary, hypothesis‐generating insights. Only a subset of tentatively identified compounds was included, and the selected bacterial receptors were chosen based on mechanistic plausibility, focusing on targets central to cell‐wall synthesis and DNA replication (Spencer and Panda 2023; Dabhi et al. 2024; Kota et al. 2014; Elsewedy et al. 2024). While the docking results suggest possible interactions between certain mushroom‐derived compounds and bacterial targets, they do not establish causality or confirm that these molecules are solely responsible for the experimentally observed antimicrobial activity. Instead, these findings should guide future work aimed at isolating, characterizing, and experimentally validating candidate bioactive compounds.

## 5 Conclusion

*Pleurotus ostreatus* showed a nutritionally relevant composition and measurable but limited in vitro antibacterial activity, with the ethanolic extract outperforming the aqueous extract under the tested conditions. The study is important because it links food composition, crude‐extract antimicrobial activity, tentative chemical profiling, preliminary safety assessment, and exploratory docking in one context‐specific dataset. These findings support the potential role of oyster mushroom as a nutrient‐contributing food material and as a source of candidate bioactive compounds for future food‐quality and functional‐food research. However, the maternal and child health relevance should be interpreted cautiously: the present study provides laboratory and compositional evidence only, and future work should evaluate nutrient bioavailability, standardized extraction, purified compounds, georeferenced sampling, solvent controls, in vivo safety, and dietary‐intervention outcomes before health claims are made.

## Author Contributions

**Abdirasak Sharif Ali:** conceptualization, writing – original draft, investigation, formal analysis, visualization, writing – review and editing, supervision, methodology. **Yahye Ahmed Nageye:** writing – original draft, writing – review and editing. **Kizito Eneye Bello:** conceptualization, writing – original draft, methodology, writing – review and editing, validation, formal analysis.

## Funding

The authors have nothing to report.

## Ethics Statement

Clinical bacterial isolates were retrieved from anonymized laboratory collections in accordance with applicable institutional procedures; no human subjects were directly enrolled, and no patient‐identifying information was used.

## Conflicts of Interest

The authors declare no conflicts of interest.

## Data Availability

Data will be provided upon request from the corresponding author.
